# Communication between Norwegian Pakistani patients and healthcare providers about traditional and complementary medicine: a qualitative study

**DOI:** 10.1080/17482631.2025.2579389

**Published:** 2025-11-14

**Authors:** Saliha Khalid, Trine Stub, Agnete Egilsdatter Kristoffersen, Christine Råheim Borge, Lise-Merete Alpers

**Affiliations:** a The National Research Center in Complementary and Alternative Medicine (NAFKAM), Department of Community Medicine, Faculty of Health Sciences, UiT The Arctic University of Norway, Tromsø, Norway; b Lovisenberg Diaconal Hospital, Oslo, Norway; c VID Specialized University, Oslo, Norway

**Keywords:** Pakistani immigrants, traditional medicine, complementary medicine, cultural competence, patient centered communication

## Abstract

**Purpose:**

Norwegian Pakistanis use traditional and complementary medicines (T&CM) to promote well-being and treat illnesses. Effective communication between T&CM users and healthcare providers (HCPs) about these practices is essential for patient safety. Therefore, this study aimed to explore HCPs’ experiences in discussing T&CM practices with Norwegian Pakistani patients.

**Methods:**

Four focus group interviews and one individual in-depth interview were conducted with HCPs in Oslo, Norway, between May and October 2024. Braun and Clarke’s reflexive thematic analysis (RTA) was used to generate themes highlighting the shared meanings of the participants’ experiences.

**Results:**

Two main themes were generated: 1) Complex consultations and 2) Enhancing communication by modifying attitudes, knowledge, and practices. Consultations with immigrant patients were complex due to language barriers, participants’ limited knowledge of T&CM, and various patient-related obstacles. Despite these challenges, participants demonstrated a strong motivation to overcome them. Improving HCPs’ knowledge about T&CM and demonstrating openness and interest in what matters to patients can build trust and promote effective communication about T&CM.

**Conclusion:**

HCPs lack knowledge about T&CM used by Norwegian Pakistani patients and rarely communicate about this topic in consultations. The results show an urgent need to promote measures that enhance communication for patient safety.

## Introduction

According to the World Health Organization (WHO), it is a fundamental right of every human being to attain the highest standard of health and to receive equal healthcare services regardless of their religion, culture, or ethnicity (Human rights, 2023). This universal right often encounters practical challenges in multicultural settings as migration introduces complex language, cultural, and communication barriers (Ramzan et al., 2017; Suphanchaimat et al., 2015). When people migrate from one country to another, they bring their cultural values and beliefs regarding health, illness (Håkonsen & Toverud, 2011), and traditional healing practices and continue these practices regardless of the prevailing healthcare system in the new country (Pieroni et al., 2008). Due to cultural diversity and diverse expectations, ineffective communication between HCPs and patients can lead to negative healthcare experiences for immigrant patients (Suurmond et al., 2011). It also poses a substantial barrier to global health equity (Napier et al., 2014). Therefore, it is important for HCPs to have knowledge about immigrant patients' cultural practices and beliefs, including T&CM, to effectively communicate about these topics (Park & Ahmed, 2023; Radl-Karimi et al., 2020).

Traditional medicine is known as *“the knowledge, skills, and practices based on the theories, beliefs, and experiences indigenous to different cultures, whether explicable or not, used in the maintenance of health as well as in the prevention, diagnosis, improvement, or treatment of physical and mental illness”* (WHO traditional medicine strategy, 2014−2023, 2013). In some countries, traditional medicine is used interchangeably with complementary medicine (World Health Organization, n.d.), which refers to healthcare practices that are not part of a country’s own tradition nor part of conventional medicine, and are not fully integrated into the dominant healthcare system WHO traditional medicine strategy: 2014−2023, (2013). T&CM merges the terms traditional medicine and complementary medicine and includes practices (e.g., herbal medicine, spiritual and mind–body therapies), products (e.g., herbs, animal–based products, and minerals), and provider–based therapies (e.g., traditional healers, homeopaths, acupuncturists) (WHO traditional medicine strategy: 2014−2023, 2013). Generally, immigrants and ethnic minority groups are more likely to use T&CM than the majority population (Agu et al., 2019; Elewonibi & BeLue, 2016). Therefore, it is essential to communicate about these practices during consultations.

Patient–centered communication enhances the sense of partnership in the HCP–patient relationship (King & Hoppe, 2013). It enables HCPs to build trust, express empathy, understand the patient’s perspectives of illness and treatment, and improve the quality of the HCP–patient relationship (Hashim, 2017; King & Hoppe, 2013). Kringlen and Finset also emphasized the importance of maintaining a strong HCP–patient relationship as an essential component of clinical consultations (Kringlen & Finset, 2006). However, research shows a lack of communication between HCPs and their patients regarding patients’ use of T&CM (Foley et al., 2019; Shelley et al., 2009). Due to cultural differences and different health and illness beliefs, such communication becomes more challenging for HCPs when the patients are immigrants. In addition, the language barrier is the most prevalent challenge in delivering healthcare services to immigrant patients in high–income countries (Brandenberger et al., 2019). A systematic review of the experiences of South Asian immigrants in the West regarding patient–centered care identified inadequate HCP–patient communication as the main factor contributing to poor patient–centered care (Vakil et al., 2023). The review emphasized that addressing the communication barriers between immigrant patients and HCPs can improve patient experiences (Vakil et al., 2023).

Pakistanis are one of the largest non–Western ethnic minority groups in Norway (Immigrants and Norwegian-Born to Immigrant Parents, by Immigration Category, Country Background, Contents and Year, 2025) and experience health challenges due to various chronic diseases, including diabetes, cardiovascular diseases, and psychological distress (Rabanal et al., 2015; Syed et al., 2006). T&CM is extensively used in Pakistan (Anwar et al., 2012; Anwar et al., 2015), and Pakistani immigrants bring and use T&CM after migrating to other countries (Pieroni et al., 2008; Ramzan et al., 2017). A study exploring traditional medicine among Pakistani immigrants in Norway found the use of various food items, herbs, animal products, and religious rituals for treating illnesses (Khalid et al., 2024). In Pakistan, fungal contamination of medicinal plants (Ahmad et al., 2014) and toxic heavy metals in traditional herbal medicines have been reported (Anwar et al., 2024). Moreover, T&CM can negatively interact with conventional treatment, posing direct risks to patients (Asher et al., 2017; Fisher et al., 2002). Poor communication between HCPs and T&CM users about these modalities can also indirectly affect the HCP–patient relationship, patient satisfaction, and compliance with treatment (Wardle & Adams, 2014). Given these findings, it is imperative for HCPs to be well–informed about these practices and to discuss them with patients to ensure their safety.c

The literature review above demonstrates the lack of knowledge about the HCPs' experiences regarding communication and understanding of T&CM. Thus, this study aimed to assess HCPs' knowledge about T&CM use among Norwegian Pakistani patients and to explore their communication experiences about these modalities in consultations with this patient group.

### Methodology

A qualitative, descriptive, and interpretive design was used (Clarke & Braun, 2022; Patton, 2014). We used an inductive approach to RTA and conducted four focus group interviews and one individual in–depth interview from May to October 2024. Focus group interviews with multidisciplinary HCPs facilitated social interaction and produced high–quality data due to group dynamics. It helped us to delve into participants' experiences regarding the topic (Patton, 2014). The individual interview was conducted for practical reasons because the interviewee could not attend any focus group interviews. We used the Reflexive Thematic Analysis Reporting Guidelines by Braun and Clarke to conduct this study according to the values of RTA (Braun & Clarke, 2024).

#### Study setting and recruitment

The study was conducted at one hospital in Oslo, the capital of Norway. In 2025, 34.96% of the population in Oslo corresponds to immigrants and their children (Immigrants and Norwegian-Born to Immigrant Parents, by Immigration Category, Country Background, Contents and Year, 2025). Pakistanis are one of the largest non–Western ethnic minority groups in Norway, with 43,215 immigrants and Norwegian–born to immigrant parents in 2025 (Immigrants and Norwegian-Born to Immigrant Parents, by Immigration Category, Country Background, Contents and Year, 2025). Approximately half of them live in Oslo and its surrounding areas. Due to the large Norwegian Pakistani community in Oslo, the hospital was an ideal setting for the study. Participants from different hospital departments were approached via email and recruited using a purposive sampling strategy (Patton, 2014). The inclusion criteria were: (a) HCPs employed at the hospital, (b) more than one year of experience in clinical practice, and (c) experience treating Norwegian Pakistani patients. HCPs recently employed at the hospital with less than one year of clinical experience, as well as those without any experience treating immigrant patients, were excluded from the study. One of the authors (CRB) facilitated participant recruitment by distributing invitation letters to all departments and inviting the HCPs to participate in the study.

#### Participant characteristics

A total of 23 participants were interviewed, consisting of 22 women and one man. They included nurses (*n* = 17), clinical nutritionists (*n* = 2), medical doctors (*n* = 2), physiotherapists (*n* = 1), and pharmacists (*n* = 1). The participants had between 1 and 38 years of clinical experience. Table 1 shows the demographic and professional characteristics of the participants.

**Table 1.** The characteristics of the participants.

	Number of participants (*n*)
**Gender**	
Male	1
Female	22
**Age**	
Less than 30 years	7
30−49 years	11
50 years or more	5
**Profession**	
Nurses	17
Physicians	2
Clinical Nutritionists	2
Physiotherapists	1
Pharmacists	1
**Level of education**	
Bachelor	9
Master	14
**Years of clinical practice**	
0−10 years	11
11−20 years	7
More than 20 years	5
**Country of origin**	
Norway	18
Another Western Country	1
Non–western Country	4

#### Data collection

The interviews were conducted by the last author (LMA) and the first author (SK) between May and October 2024. One of the interviewers is a professor with a PhD and has extensive experience working with immigrant patients in Norway as a nurse. The other interviewer is a PhD student with a background in pharmacy. The interviews lasted between 55 and 96 minutes (on average, 68 minutes). Three of the focus group interviews were conducted face–to–face at the hospital. For practical reasons, one of the focus group interviews and the individual interview were conducted digitally on Teams. Before the interviews, we collected demographic information regarding gender, age, education level, profession, years of professional experience, and ethnic background. The research team developed a semi–structured interview guide, informed by a literature review, to collect data on the following topics: *experiences working with Norwegian Pakistani patients, personal opinions and knowledge about T&CM, and communication about T&CM with these patients*. The interview guide is available as supplementary material. SK pilot–tested the interview guide with two HCPs through an online focus group interview, which led to some adjustments. The feedback suggested starting the interview with more open questions and exploring the participant's general experience working with the target group. We applied the concept of information power (Malterud et al., 2016) to assess the quality and depth of the data we collected. We obtained sufficient information power after four interviews due to the prior experiences of interviewers with qualitative interviewing, the narrow study objective targeting a specific immigrant population, the good quality of the dialog due to the focus group dynamics, the particular sample having experience working with the Pakistani immigrant patients, and in–depth analysis of the participant experiences. The interviews were conducted in Norwegian at the hospital and were audio–recorded. SK took field notes during the interviews. A certified language services provider in Norway handled the transcription and translation of the interviews. The interviews were transcribed and translated into English by a professional translator. The translated files were proofread by SK for errors and were rectified accordingly.

#### Data analysis

We used Braun and Clarke’s reflexive thematic analysis (RTA) to generate patterns of participant experiences (Braun & Clarke, 2006; Clarke & Braun, 2022). SK and LMA analyzed the data, and the other co–authors participated in the discussion of the analysis. Data collection and analysis were performed iteratively, and we started coding the data before obtaining the complete data set. We followed the six–step approach developed by Braun and Clarke to conduct RTA (familiarization of data, generating codes, constructing themes, reviewing themes, defining and naming themes, and producing the report) (Braun & Clarke, 2006). Initially, SK and LMA familiarized themselves with the data by reading and re–reading the transcripts to get the essence of shared experiences. SK created written reflections after reviewing the transcripts and summarized her thoughts about the interaction between the participants. She incorporated field notes from the interviews into her reflections. SK and LMA coded the data inductively. After multiple rounds of revision and discussions with the research team, we generated two themes, each with three sub–themes. The discussions and written reflections helped develop the themes. We renamed the themes and sub–themes several times to reflect our data interpretation accurately. We used QSR–NVivo v10.0 software to organize themes and associated participant quotations effectively (Wong, 2008).

#### Reflexivity

Reflexivity in RTA must extend beyond self–reflection to include awareness of the knowledge generated and methods used in research (Braun & Clarke, 2024). The first author is a pharmacist from Pakistan who knows about Pakistani culture and T&CM. The last author and a co–author are nurses who have worked with immigrants in various settings and have extensive research experience in immigrant health. This insider perspective facilitated an understanding of the nuances of the situation. The other two co–authors have experience in research about T&CM. In addition, all authors have expertise in qualitative research. These prior experiences provided a multifaceted approach to data analysis. We engaged in an iterative data interpretation process and reflected on our perspectives, roles, and their impact on the data.

#### Ethical considerations

The Norwegian Agency for Shared Services in Education and Research (Sikt) (reference number: 447080) assessed this study. It concluded that the processing of personal data was lawful and followed the data protection legislation. The Regional Committees for Medical and Health Research Ethics (REK 493745) determined that the study did not qualify as health research in Norway and thus did not require their approval. We briefed the participants about the study’s objectives, methodology, and content. They were informed that participation was voluntary and that they could withdraw without consequences. Before starting the interviews, participants’ verbal and written informed consent was obtained. We followed UiT The Arctic University of Norway’s research data management guidelines (UiT the Arctic University of Norway, 2021). To maintain anonymity, each participant was assigned an identification number, including a digit and an alphabet. We maintained the confidentiality of the participants throughout the research process. The study followed the research standard of the Helsinki Declaration (“World Medical Association. Declaration of Helsinki: ethical principles for medical research involving human subjects,” 2013).

## Results

We identified two main themes and six sub–themes (Table 2) that highlight the challenges in communicating about T&CM with Norwegian Pakistani patients during consultations, while also suggesting solutions to improve dialog based on healthcare providers' experiences. Language barriers and limited knowledge of T&CM contributed to the complexity of consultations; however, open communication about patients' preferences can improve such discussions. These findings provide a comprehensive understanding of healthcare providers' experiences in navigating T&CM–related discussions with Norwegian Pakistani patients.

**Table 2.** Themes and sub–themes.

Themes	Sub–themes
1. Complex consultations	Language barrier in communication Lack of knowledge about T&CM T&CM use is a private matter
2. Enhancing communication by modifying attitudes, knowledge, and practices	Knowledge is the key to ask informed questions Openness, curiosity, and interest during consultations Practical strategies to improve communication

### Theme 1: complex consultations

The clinical consultations were complex due to various factors highlighted by the participants, leading to poor communication about T&CM. The language barrier was a significant factor contributing to these situations, along with the limited knowledge of T&CM among participants and patients. Moreover, some patients considered the use of T&CM a private matter, making it challenging to inform the participants about this use.

#### Subtheme: language barrier in communication

Participants discussed the language barrier as a significant challenge in communicating about T&CM, as patients often used Pakistani local names for the T&CM modalities they employed. This led to difficulties for the participants in finding scientific literature about the products and further providing the patients with valuable information upon inquiry. The language barrier added complexity to the clinical consultations, as illustrated by one of the participants: *There are no words for it [T&CM] in Norwegian, which makes it difficult to explain or understand*.* It cannot be translated accurately* (3D). A physician elaborated: *Patients bring something from their native country, which they don’t know the name of in Norwegian, and I’m unable to find out what it is* (4 C). A pharmacist explained: *Patients often come in with photos of medicines they purchased in their native countries and then ask whether we have them in Norway. It can often be challenging to understand because it’s in a different language, and we cannot translate it. It is difficult to follow up on this or help them obtain it in Norway* (4A).

Participants experienced the ineffectiveness of interpreters during consultations. They discussed that the interpreters usually have a limited understanding of medical terminology, which increases the risk of translation errors. An HCP of Asian descent, born and raised in Norway with limited proficiency in her mother tongue, shared: *I was unsure of my language as I hadn’t practiced it for a long time, so I ordered an interpreter. He tried translating treatment and medicine, but it did not work. I had to take over because he couldn’t find words* (3B). Some of the participants mentioned that they avoid asking about T&CM during consultations when an interpreter is present. The main reason was that consultations with an interpreter tend to take longer, and the standard consultation time is already quite limited. A participant stated: *We are better at asking about it [T&CM] from those who speak Norwegian well. When I use an interpreter or have language challenges, I don’t prioritize those questions [about T&CM]* (1E). The language barrier became more challenging, along with the ineffectiveness of interpreters and the short consultation times. It further complicated the consultations and prevented participants from delving into T&CM discussions with the patients.

#### Subtheme: Lack of knowledge about T&CM

The participants acknowledged their limited understanding of cultural contexts, including T&CM, as a reason for poor communication and complex consultations. They found it challenging to understand the patients' choices due to their lack of cultural knowledge. A nurse shared the story of a patient with a Pakistani background who became quite ill after chemotherapy and then chose to discontinue the treatment. It was challenging for her to understand this patient’s choice, and she stated: *I perceived him as an unusual patient who was spiritual or concerned with being in touch with oneself and nature. He was dependent on feeling that he was doing right for his body or not* (3A)*.* Due to the lack of knowledge, participants rarely initiated conversations about T&CM. A participant mentioned: *Because I don’t know the culture well, I avoid asking about it [T&CM]* (3C). Participants often directed patients asking about T&CM to other HCPs because they didn't have enough knowledge to answer their queries. A nurse stated: *I discuss it if the patient asks about it. But when the patient asks [about the adverse effects of T&CM], I explain that I don’t know. You can ask the doctor, but it is not certain that the doctor knows it either* (1A).

The participants attributed their limited understanding of T&CM to their education being focused on conventional medicine. They learned Western perspectives on health and healing, but did not learn about T&CM. A pharmacist explained: *We did not learn about T&CM in our medical training. It’s more self–taught than anything we’ve learned during our studies* (4A). The lack of knowledge was more challenging when the participants had an inflexible attitude toward their patients using T&CM. Participants related this attitude to their medical training. A participant explained their attitude: *We recommend conventional medicine, and you should cut out everything else* (3A). A nurse reported: *It probably also has something to do with our mindset. We are here to help and contribute to the field of conventional medicine. This is the mindset I have* (1F)*.*

Participants highlighted that, in addition to their lack of knowledge, some patients were unaware of the composition of the T&CM they used. Thus, it made it more challenging for the participants to discuss T&CM in such consultations. One participant observed: *The patient didn’t know what herb was in the drop she had, and she asked about using it. I don’t have much experience with it myself and find it difficult to give guidance on what to do* (1A). The participants mentioned that some patients brought T&CM products purchased from their home country to the doctor's office and provided no verbal information. Therefore, it was challenging for the participants to identify and discuss those products. A physician explained: *Sometimes, we recognize the medicines patients bring with them. However, at other times, we are unsure of their contents* (5A). Thus, the lack of knowledge among participants and patients created a barrier to open discussions about T&CM, resulting in more complex consultations.

#### Subtheme: T&CM use is a private matter

The participants experienced that patients did not disclose their use of T&CM because they considered it a private matter. T&CM use is rooted in the patient's cultural background and should remain confidential. A participant stated: *For some* patients*, using T&CM is private, something they do for themselves and prefer not to discuss openly* (2A). Another added: *I don’t know why they never talk about it. They consider it very private* (3A). Thus, considering the use of T&CM to be a private matter, it wasn't easy to bring it up in the consultations. In line with this, a physician added: *Some patients don’t like it when we ask about their diets. They may think we’re prejudiced* (5A). Participants mentioned that disclosing information about T&CM was not important for the patient, as they viewed it as a routine aspect of care. Participants also experienced that some of their patients were afraid to share and didn’t consider it appropriate to be open about using T&CM. A participant stated, *I’ve come across a patient who attended a consultation and said*, “Oh, I don’t know, should I say it or not, but I’m taking a drop.” *So, they are a bit skeptical about our response* (1A).

The language barrier in consultations, combined with participants' limited knowledge of T&CM, hindered effective communication about T&CM. Additionally, the patient’s perception of T&CM as a private matter further complicated this issue and led to complex consultations.

### Theme 2: Enhancing communication by modifying attitudes, knowledge, and practices

While discussing the perceived barriers to effective communication about T&CM, participants expressed motivation to overcome these obstacles and identified several factors that could facilitate this process. They debated the importance of being knowledgeable about T&CM, showing interest, and being open, humble, and curious about what is essential for the patients. They discussed how this could lead to trust building and make patients feel involved in designing their treatment plans.

#### Subtheme: Knowledge is the key to ask informed questions

Participants expressed that having knowledge about T&CM and being aware of the immigrant patient’s culture is essential to communicating effectively about their T&CM use. A participant explained what culture in this context could entail: *It may contain everything from the weather to clothing. What do they like? What are they eating? What do they believe in Asia? What type of advice are they getting from family and friends? Do they follow this advice? If they grew up here, what was passed on to them by their parents?* (4B). This preliminary knowledge was necessary to ask specific questions and to have meaningful conversations about T&CM. An endocrinologist inquired about the diets of diabetic patients and mentioned how knowledge of patients' eating habits can contribute to asking culturally tailored questions. *If someone is from Pakistan, I would ask whether they eat chapati *[made from wheat flour]* and rice. Some people eat a lot of dates, which contain concentrated sugar. You must ask them about this specifically: “Do you eat dates? Do you eat figs?”* (4C). She used social media to understand her patients' views on the disease and its treatment, and added: *I use Instagram to keep up with the latest trends. Many patients have firm opinions about what they use, its effect, or how well or poorly it works. So, I have to be able to comment on it* (4C).

Some of the participants were aware of the safety concerns associated with T&CM, which motivated them to inquire about its use, with a specific focus on interactions with conventional medicine. A physician stated: *I’m very focused on the safety of medicine. We repeatedly find that if we do a poor job with this [communicating about T&CM], it will have consequences for the patient's treatment later on* (4C). Participants' knowledge and experience about the adverse effects of T&CM led to specific questions about it. A physician added: W*e have had someone who had Vitamin D toxicity. After that, we are more direct when asking about it* (5A). The participants gathered information about T&CM safety and drug–herb interactions from Google, UpToDate, medical databases (PubMed), clinical pharmacists, and RELIS (Regional Medicines Information and Pharmacovigilance Centers), as illustrated by this participant: *RELIS is the regional medicine information center. You can send them all the suspected adverse effects, and they will evaluate them considering the medicinal products we recommend for the patients (*5A). Thus, participants' knowledge of T&CM, medical safety, and patients' culture enabled them to ask culturally tailored questions about T&CM.

#### Subtheme: Openness, curiosity, and interest during consultations

While discussing the measures to promote communication about T&CM with immigrant patients, participants highlighted the need to increase awareness of one’s attitudes and modify behaviors during consultations. Openness and curiosity were considered essential to obtaining information about T&CM. A nurse explained: *If patients know that we will still say that conventional medicine applies, then there is no point for them to talk about it [T&CM]* (1F). Participants considered their curiosity and openness to be essential during consultations for building patient trust. A participant said: *If they tell you about something they have used, you can search online for the content of the product. It may increase their trust, and they might share more* (1C). Another nurse explained how openness and humility may facilitate good relationships with patients. *Being open and humble is important because if you say they should stop it [T&CM], they will not believe in what we do* (3C).

Participants who were familiar with T&CM tended to be more open regarding patients’ use of T&CM. One participant mentioned: *Before I say it is irrelevant, I try to understand the motivation for using these products* (3B). A clinical nutritionist supported the use of T&CM for patients with inflammatory bowel syndrome, as it provided symptomatic relief alongside conventional treatments. She also had experience using T&CM from her culture and stated: *If they find that T&CM helps and has a placebo or real effect, we have reached the treatment goal. I try to meet them open–minded, thinking that a lot can help and work* (1C). Another participant emphasized the importance of integrating T&CM products into patients’ treatment plans and stated: *If we give Western medical advice, we can still respect and understand their thinking and try to weave it together* (1B).

Some of the participants shared their experiences of how they managed to remain open in clinical practice. A physician (4C) asked her patients about any non–conventional treatments they are using, including supplements or remedies obtained from others or purchased online, and inquired about their sources. She emphasized inquiring about T&CM use specifically from each patient, as patients from the same culture can vary widely in their practices. In addition, she asked her patients to take pictures of the T&CM they used, which she found very helpful for identification. During consultations with an interpreter, she asked the patients to spell out the T&CM modality they used in their native language, which helped the interpreter identify it. Respecting and acknowledging the patient’s personal and cultural preferences can foster a good patient–provider relationship and enhance communication about T&CM.

#### Subtheme: Practical strategies to improve communication

The participants discussed practical strategies for solving the challenges associated with complex consultations. Some shared their experiences about what worked for them in practice to encourage such communication, while others suggested strategies that could work in their specific context.

One suggestion was to develop an evidence–based list of T&CM products used by specific patient groups, including their names, indications, interactions, and adverse effects. Participants suggested using this list as a guide or reference in consultations and considered it a time–efficient method. A clinical nutritionist suggested: *It is great to have a form with all the most common modalities [T&CM] and an explanation of the reason for use. It is also essential to know whether a specific T&CM modality can have any effect, is harmless, or interacts with other substances. It can result in a better understanding, making it easier to discuss and provide reasonable answers instead of saying “I don’t know”* (1D). Other participants (1E, 1F, 1A, 3A, 3B) suggested tailoring this list for different patient groups. They recommended including Norwegian and Pakistani names of the T&CM modalities, along with their images, to help HCPs identify the correct T&CM in cases of language barriers.

Another strategy was to create awareness of this topic among the hospital staff through lectures and discussions, as stated by one of the participants: *It’s important to raise awareness about T&CM, as we’ve done now. It is a good start* (3C)*.* Participants (1C, 1D, 2A, 2G, 3B, 4C, 5A, and 3A) shared various examples of T&CM that their patients used, including lemon water, garlic, hot water, olive oil, turmeric, fennel seeds, cinnamon, ginger, cloves, and honey. The knowledge about T&CM shared during the interviews enabled participants to understand their non–Western patients and encouraged them to learn about their patients’ T&CM preferences. A nurse stated: *I have had two patients who were very keen on drinking hot water, and I never knew why. However, I know it now and have a different attitude toward it* (3A).

Collaborating with other HCPs was also considered essential, as stated by a nurse: *There may be modalities that interact with conventional treatment, which I knew little about. Then, I informed the physician to address this during the next consultation* (4B)*.* A participant explained how pharmacists were a source of information about T&CM used by their patients. *Some patients wonder if they can use T&CM in combination with conventional treatments. I can search for interactions without finding anything, but pharmacists can access several platforms and often do a good job for us* (2B). Some participants (3B, 3D, 4D, 1B) considered HCPs with immigrant backgrounds a resource for their ethnic Norwegian colleagues. A participant shared her experiences working with a nutritionist of Pakistani background and stated: *It created enormous trust when patients were talking to someone who understood and respected them. Then, I can easily perform my tasks and discuss activities as a physiotherapist* (1B). A physician (4C) also highlighted the importance of cultural interpreters in this arena.

The participants were motivated to solve the challenge of complex consultations and to improve communication about T&CM with immigrant patients. They suggested strategies such as searching for information, showing interest, talking about safety and interactions, and drawing on experiences from their non–Western colleagues.

## Discussion

This study aimed to explore the experiences of HCPs in communicating about T&CM with Norwegian Pakistani patients. The participants found consultations with these patients complex and challenging due to several factors. A significant issue was the language barrier, which included a lack of common terminology and understanding of the specific T&CM products used by patients. Additionally, patients' hesitation in disclosing their use of T&CM further complicated the consultations. Demonstrating curiosity, openness, and interest in patients' T&CM practices was understood as crucial. Therefore, establishing trustful relationships with patients and enhancing HCPs' knowledge of T&CM was essential.

### Complex consultations require cultural competence

A clinical consultation aims to assess a patient's health needs and develop a personalized treatment plan while providing education and support. The patient–centered clinical method of consultation involves exploring a patient's experiences of health and illness, understanding the whole person, and finding common ground for mutual decisions, thus enhancing the HCP–patient relationship (Stewart et al., 2014). According to the participants, the consultations with Pakistani immigrant patients were challenging due to language barriers, cultural differences, different understandings of illness and health, and different attitudes among HCPs and patients. These results align with previous research (Barrio‐Ruiz et al., 2024; Kavukcu & Altıntaş, 2019; Priebe et al., 2011; Suphanchaimat et al., 2015). Other barriers highlighted by our study were time constraints in consultations, the ineffectiveness of interpreters, and a lack of HCPs’ knowledge about the culture, including T&CM, which are well–known in the literature (Alkhaled et al., 2022; Balneaves et al., 2022; Hall et al., 2018; Hanssen & Alpers, 2010; Vandecasteele et al., 2024). Our findings emphasize the importance of demonstrating open and non–judgmental interest during consultations, which can also improve HCPs’ understanding of the topic (Epstein et al., 2005; Shelley et al., 2009).

It is essential that HCPs have some knowledge about the patient’s culture to collect information about T&CM use. *Cultural knowledge* encompasses understanding the patient’s historical, geographical, and socio–cultural background, as well as their perceptions of health and illness, and cultural beliefs, values, and practices (Campinha-Bacote, 2002). It is the cornerstone of patient–centered communication about T&CM (Hunter et al., 2021). According to a Norwegian study, nurses report having inadequate knowledge about non–Western philosophies of disease and treatment (Alpers & Hanssen, 2014). Another study emphasized the inadequate cultural understanding of interpreters in the Norwegian healthcare system and HCPs (Hanssen & Alpers, 2010). This lack of knowledge could be a reason for HCPs' unsupportive attitude towards T&CM (Aizuddin et al., 2022). Our findings indicate that a lack of cultural understanding results in inadequate communication about T&CM, underscoring the importance of enhancing HCPs' knowledge on the topic. On the other hand, knowing all cultures is not possible and practical, considering the multicultural society, making it a significant challenge for the HCPs working with immigrant patients. Some participants used various sources to fulfill their information needs about T&CM, like UpToDate, which was also reported in another study conducted in Norway (Stub et al., 2018).

During medical education, HCPs are generally not trained to discuss culture–related topics with patients, such as the use of T&CM (Jha et al., 2015; Vandecasteele et al., 2024). Participants in this study agreed that they did not receive training on how to discuss their T&CM use with patients. This lack of training was perceived as a barrier to communicating about T&CM. Therefore, the participants emphasized the importance of being more positive and open to alternative forms of treatment. This aligns with a study by Alpers and Hanssen, which underlined the need for in–service education and training about different forms of therapy for Norwegian HCPs (Alpers & Hanssen, 2014). Education can equip HCPs with essential communication skills and a deeper understanding of patients’ non–conventional therapies and practices (Hunter et al., 2021).

Research shows that T&CM users usually do not disclose the use of T&CM to HCPs (Foley et al., 2019). The participants mentioned that patients perceive disclosure as unimportant, which can be one of the reasons for non–disclosure. It has been highlighted previously (Foley et al., 2019). This patient behavior can be related to the quality of the HCP–patient relationship and communication style. A meta–analysis suggests that a poor HCP–patient relationship is associated with the disclosure of T&CM use (Foley et al., 2019). Another significant reason for non–disclosure is the lack of inquiry from HCPs (Foley et al., 2019; Shelley et al., 2009), which can be associated with HCPs' lack of knowledge about T&CM and culture (Balneaves et al., 2022; Corina et al., 2016). Many of our participants didn’t ask patients about T&CM use because they were unaware that T&CM is extensively used in their patients’ culture. A literature review pointed out that the HCPs only discuss T&CM upon patient inquiry in consultations (Stub et al., 2016). Therefore, it is essential that the HCPs take the initiative and responsibility to raise these issues in consultations (Shelley et al., 2009; Stub et al., 2017). This aligns with studies indicating that HCPs should initiate such discussion, as patients often hesitate due to fear of HCP’s negative reactions (Balneaves et al., 2022).

Kleinman also recommends that HCPs actively seek information about patient preferences regarding the use of healthcare services (Kleinman, 1980). Kleinman's model of local healthcare systems comprises three overlapping components: popular (encompassing individual, family, social, and community beliefs and practices), professional (conventional medicine), and folk (T&CM providers) sectors (Kleinman, 1980). The content and impact of each sector vary across cultures, and patients transition between them throughout an illness. Being aware of these transitions and communicating openly about them is essential for HCPs (Kleinman, 1980). Moreover, it is essential to consider intracultural variations because when individuals from a cultural group are presumed to share the same cultural values, attitudes, beliefs, or practices, stereotypes persist. Therefore, following a specific pattern to interact with any particular group is ineffective because each patient’s culture is complex and ever–changing, and it cannot be solely defined by race or language group (Devillé et al., 2011).

Thus, improving the cultural competence of HCPs is essential for addressing the challenges of complex consultations with immigrant patients and fostering effective communication about T&CM.

### Practical measures to enhance communication

Participants highlighted practical strategies to ensure effective communication, including developing a list of T&CM products, providing educational opportunities about T&CM for HCPs, and interprofessional collaboration. Many of our findings align with studies to understand the experiences of HCPs regarding healthcare for immigrants (Hjörleifsson et al., 2018; Priebe et al., 2011). A review has also highlighted the need for better training opportunities for HCPs to improve their cultural knowledge so that they can better address the specific needs of immigrants (Allegri et al., 2025). Education can provide HCPs with essential communication skills and enhance their understanding of T&CM therapies and practices utilized by patients (Hunter et al., 2021). To the best of our knowledge, the literature has not highlighted the development of a T&CM list with images for use as a reference in consultations. This strategy may be an effective way to enhance communication, particularly in consultations where language barriers exist. The first step towards this strategy is to collect evidence–based information about T&CM that is extensively used by the target population.

Interprofessional collaboration among HCPs was considered essential by the participants to enhance communication about T&CM. It plays an important role in providing patient–centered care (Hunter et al., 2021), and various interrelated factors influence it, including medical dominance, clarity of HCPs' roles, shared vision, and education (Nguyen et al., 2019). Another study highlighted nurses' role in communicating with physicians about the patient’s T&CM use (Hall et al., 2018). In addition, collaborating with HCPs from non–Western backgrounds was also highlighted. Research indicates that immigrant general practitioners in Norway have a higher proportion of immigrant patients compared to native Norwegian general practitioners (Diaz et al., 2014). Participants mentioned that immigrant HCPs possess the cultural knowledge and skills to communicate about T&CM. This aligns with a study highlighting that immigrant general practitioners in Norway demonstrate cultural competence during consultations (Díaz and Hjörleifsson, 2011). They leveraged their international expertise and personal experiences from their home countries to support particular patients, serving as a distinctive asset in clinical practice (Díaz and Hjörleifsson, 2011). This expertise of immigrant HCPs can benefit other HCPs in understanding the preferences of immigrant patients.

Another strategy to enhance communication about T&CM was offering cultural interpreters in healthcare settings. Participants highlighted the current unavailability of this service, which is also limited to other European countries (Jaeger et al., 2019). Moreover, there are various barriers faced by HCPs and immigrant patients in using the interpreter services that are currently available in the European healthcare systems (Vange et al., 2024). Another study conducted in Norway underlined the need for qualified and legal cultural interpreters (Hanssen & Alpers, 2010). These interpreters can bridge the linguistic and cultural gaps between HCPs and immigrant patients (Verrept, 2019), leading to culturally sensitive care (Devillé et al., 2011). They can enhance the quality of clinical care (Brown et al., 2021; Karliner et al., 2007) and improve patient and provider satisfaction (Heath et al., 2023). On the other hand, when interpreters take on cultural roles, their responsibilities and discretion expand, potentially blurring the lines of accountability between them and HCPs (Alpers, 2017). Therefore, using cultural interpreters can be challenging and requires clear role descriptions to ensure effective collaboration.

These measures can facilitate HCPs in providing culturally sensitive healthcare (McGough et al., 2022). It ensures that patients feel heard and understood by their HCPs, which enables them to openly discuss their health concerns, beliefs, values, preferences, and cultural practices (Bresnahan & Zhuang, 2024).

## Strengths and limitations

A significant strength of this study lies in its methodology. Using RTA facilitated a thorough understanding of the participant discussions (Clarke & Braun, 2022). Many participants had extensive experience working with immigrant patients, as the study was conducted in Oslo, where most immigrants reside. The study setting is one of the few hospitals in Norway with this expertise. The participants had different roles in the healthcare system (physiotherapist, doctors, nurses, pharmacist, clinical nutritionist), so their unique experiences added to the depth of the study. Additionally, they had diverse cultural backgrounds, encompassing different perspectives on the topic. The participants reflected on their perspectives regarding T&CM in relation to other participants and within the context of the healthcare system. This group dynamic enabled us to gather rich data (Patton, 2014). The sufficient number of participants in each group interview (*n* = 4 to 7) led to rich discussions (Patton, 2014). Three co–authors, each with different expertise, finalized the results to ensure the credibility of the research findings. The subjectivity of each researcher was a strength that added to the richness of data interpretation. Awareness of our professional roles, prior experiences, self–questioning, and informal discussions with peers enabled us to be reflexive throughout the research process. This reflective approach helped us validate our positions and understand their impact on the research process. Moreover, all research decisions and data analysis steps were recorded to ensure dependability (reliability) in the study.

The results should be considered in light of their limitations. It is not possible to generalize the study findings because of contextualization. We tried to maintain our focus on Norwegian–Pakistani patients during the interviews. However, we believe that the findings of our study might be transferable to other immigrant populations in Norway and similar settings. We tried to be as explicit as possible about the research setting and participants while ensuring their confidentiality. The individual interview was conducted as an audio session, which prevented us from observing and interpreting the interviewee's body language. There was an uneven gender distribution among participants. Future research on this topic should aim for a more balanced gender representation. Most of the participants were nurses, so participants from other professions might have presented different perspectives. Moreover, integrating patients' perspectives would have added depth to this study (Asmal et al., 2022).

## Implications for research and practice

This study highlights the need to improve the HCPs' knowledge about T&CM through education and training. By showing openness and interest in patients' use of T&CM during consultations, HCPs can strengthen their relationships with patients, encouraging open discussions and effective communication. Training opportunities should be provided to improve HCPs' communication skills, particularly when interacting with immigrant patients. Lectures and seminars can improve HCPs' knowledge of the topic. Additionally, consulting non–Western colleagues can be beneficial for addressing questions about T&CM and cultural practices.

It is important to conduct similar studies among other immigrant populations in Norway to understand communication challenges related to their T&CM practices. We also emphasize understanding the perspectives of immigrant patients on the topic and what motivates them to engage in such discussions. Future research should investigate the effectiveness of the identified strategies (e.g., developing a T&CM list) when applied in practice. For patient safety, it is essential to explore the active components and dosages of T&CM used by immigrants and their potential interactions with Western medicine.

## Conclusion

The participants had limited knowledge of traditional and complementary medicine, and rarely inquired about its use from immigrant patients. They found the topic challenging to discuss, making these consultations complex. This difficulty was partly due to language barriers and the perception that patients viewed the use of traditional and complementary medicine as a private matter. Assistance from colleagues with immigrant backgrounds can facilitate communication about the topic. For safety reasons, healthcare providers should maintain an open and curious attitude when engaging with this patient group.

## Supplementary Material

Supplementary Material Supplementary Material.

## Data Availability

The participants in this study did not provide consent for public sharing of their data. So, the supporting data is unavailable due to ethical and privacy concerns.
