# Supplementary material for: Communication between Norwegian Pakistani patients and healthcare providers about traditional and complementary medicine: a qualitative study
Source: Int J Qual Stud Health Well-being. 2025 Nov 14;20(1):2579389. doi: 10.1080/17482631.2025.2579389 (PMC12621328; doi:10.1080/17482631.2025.2579389)
Supplement: Supplementary Material [file ZQHW_A_2579389_SM5632.docx]

***Interview Guide***

| ***General Perspectives and Experiences*** |
| --- |
| - Please describe your general experience with patients from immigrant communities. - Can you share some experiences with immigrant patients who use therapies other than conventional medicine for their health? - Can you share any examples of modalities your patients from Pakistan have used for their health, aside from conventional medicine? - What are your personal/professional attitudes towards using things other than conventional medicine for health? - How do your perspectives influence your approach to patients who use such treatment methods? |
| ***Communication about T&CM*** |
| - To what extent do you discuss the use of TCM with your patients? - What do you discuss about this topic with patients from immigrant backgrounds? - How do you address these topics? - What challenges do you experience in such conversations? - What do you do to overcome these challenges? |
| ***Knowledge about T&CM*** |
| - What do you know about T&CM and their safety? - How do you obtain information about T&CM if you need it? - What do you need to handle such conversations better? - What type of information would you like about T&CM? - Is there anything else you want to add that we have not covered? |
